# Immunonutrition reduces complications rate and length of stay after laparoscopic total gastrectomy: a single unit retrospective study

**DOI:** 10.1007/s12672-022-00490-5

**Published:** 2022-07-11

**Authors:** Marzia Franceschilli, Leandro Siragusa, Valeria Usai, Sirvjo Dhimolea, Brunella Pirozzi, Simone Sibio, Sara Di Carlo

**Affiliations:** 1 Department of Surgery, University of Roma “Tor Vergata”, Viale Oxford, 81, 00133 Rome, Italy; 2 Department of Surgery “Pietro Valdoni”, University “Sapienza” of Rome, Viale del Policlinico, Rome, Italy

**Keywords:** Immunonutrition, Gastric cancer, Total gastrectomy, Upper GI surgery, Laparoscopy

## Abstract

**Background:**

Preoperative immunonutrition (IN) reduces the incidence of postoperative complications in malnourished patients undergoing upper gastrointestinal surgery. However, its effect in norm-nourished patients remains unclear. Furthermore, patients with gastric cancer undergoing laparoscopic total gastrectomy (LTG) are not routinely included in protocols of enhanced recovery after surgery (ERAS).

**Objective:**

The aim of this study was to investigate the effects of perioperative IN in patients undergoing laparoscopic total gastrectomy (LTG) within an established ERAS pathway.

**Methods:**

A comparative retrospective study of patients undergoing LTG, receiving an immune-enhancing feed plus maltodextrin load the day of surgery (Group A) versus patients who had the same operation but no IN nor fast track management (group B).

**Results:**

There were no significant differences in patient demographic characteristics between the two groups but the medium age of patients in group A was older. Thirty-days postoperative complications were respectively 8.7% in Group A and 33.3% in Group B (p 0.04). Mean and median LOS for Group A and B were also significantly different: 7.2 ± 4.4 vs 10.3 ± 5.4 and 7 vs 10 days respectively.

**Conclusion:**

Preoperative IN associated with ERAS protocol in normo-nourished patient undergoing LTG seems to reduce postoperative complications. Reduction in LOS is possibly associated to the ERAS protocol.

*Clinical trial registration* Clinical trials.gov: NCT05259488

## Introduction

Gastric cancer is the third leading cause of cancer related mortality worldwide. Treatment is multidisciplinary with surgical resection being the only potentially curative treatment [1]. Post-surgical complications are still high and mortality rate is up to 4%, despite advancements in surgical technique and perioperative care [2].

**Fig. 1:**
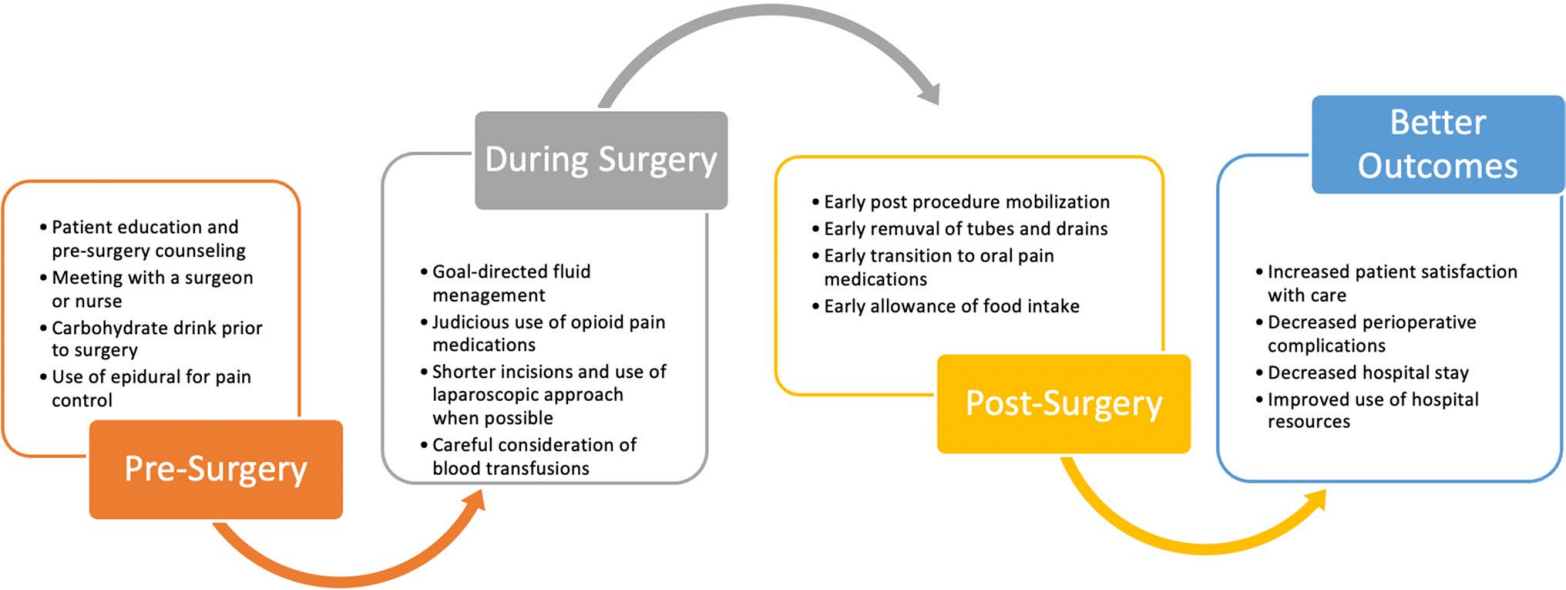
Summary of ERAS features

Enhanced recovery after surgery (ERAS) protocols, reported for gastric surgery in 2014, have proven valuable in decreasing surgical stress, improving overall outcomes and reducing the length of stay (LOS) [3]. Evidence-based guidelines focus on preoperative counselling, optimization of nutritional status and standardization of analgesia with reduction opioids and IV fluids. Laparoscopic surgery is desirable and so is early ambulation and discharge. The aim of ERAS perioperative care is the reduction of body’s physiological catabolic response to surgical stress, mediated by various stress hormones and inflammatory cytokines [4, 5] (Fig. 1).

In this context, perioperative immunonutrition (IN) is able to modulate the systemic inflammatory response by improving nutritional status and by influencing host immune response. IN is reported to decrease the incidence of postoperative infectious complications and LOS after major gastrointestinal surgery [6, 7]. However, although perioperative IN seems to reduce the rate of infective complications in malnourished patients undergoing gastrectomy, present evidences are insufficient to support a routinely use in normo-nourished patients [3, 8, 9].

The primary end-point of this study, was to compare IN within an ERAS program vs standard care in norm-nourished patients undergoing laparoscopic total gastrectomy (LTG) for advanced gastric cancer in a single unit. As secondary end-points, differences in LOS and postoperative outcomes were evaluated.

## Material and methods

### Study design and protocol

A single-center retrospective, non-parallel, comparative study.

All consecutive patients eligible for LTG for gastric cancer between November 2018 and December 2021 who meet the inclusion criteria were enrolled in the study protocol and treated with 5 days of an immune-enhancing feed plus maltodextrin load the day of surgery (Group A).

Patients and outcomes for Group A were compared with an historical control group consisting of patients undergoing LTG between January 2014 and December 2017 and treated following standard perioperative care. Data were registered on a prospectively maintained database, recording continuous and discrete variables regarding biometric data, patient-related risk factors, preoperative blood tests, tumor characteristics, neoadjuvant therapy and postoperative outcomes.

All patients had the same diagnostic work-up (endoscopy with biopsy, computed tomography, staging laparoscopy), were staged according to 8th edition of AJCC staging system and discussed in a multidisciplinary setting [10]. Neoadjuvant chemotherapy with MAGIC or FLOT regimen was given to all patients with positive lymph nodes or in case of T3/T4 N0 patients with ECOG 0–1.

Nutritional status was evaluated using the Mini Nutritional Assessment-Short form (MNA®-SF) [11]. Patients with MNA-SF score > 12 were considered normo-nourished.

IN consisted of five days of enteral nutrition with arginine + nucleotides + ω-3 fatty acids before surgery for a total of 25–30 kcal/kg/daily. Water-diluted maltodextrins (50 to 100 gr) were given two to four hours before surgery. Patients from control group did not receive nutritional support.

All patients in the two groups underwent a total laparoscopic procedure with five port technique, D2 dissection and Roux-en-Y reconstruction with side-to-side anastomoses.

All Patients in group A were treated according to ERAS 2014 guidelines [3].

Any complication (intended as any adverse event occurring during the 30-days postoperative period) including anastomotic leak, abdominal collection, bleeding, pulmonary complications (clinical symptoms, confirmed by radiological examination), surgical site infections (SSI, defined according to the Centre for Disease Control and Prevention, CDC/NHNS), was recorded and graded according to Clavien-Dindo classification [12, 13].

### Inclusion and exclusion criteria

All patients aged above 18 years with a diagnosis of stage I-III gastric cancer eligible for laparoscopic D2 total gastrectomy. Exclusion criteria were acquired or congenital immunodeficiency, previous gastric surgery, malnutrition (MNA-SF < 12), preoperative infections, ASA score > 3, emergency setting, intraoperative evidence of paraaortic node involvement, distant metastasis or peritoneal spread, conversion to open surgery.

Inclusion and exclusion criteria are summarized in Table 1.

**Table 1:** Inclusion and exclusion criteria

**Inclusion criteria**
Age > 18 years
Primary gastric cancer
Preoperative staging I–III
Eligible for laparoscopic D2 total gastrectomy
**Exclusion criteria**
Acquired or congenital immunodeficiency
Malnutrition (MNA-SF score < 12)
Preoperative infection
Previous gastric surgery
ASA IV
Emergency setting
Para-aortic node involvement
Intraoperative evidence of distant metastasis or peritoneal carcinosis
Conversion to open surgery

### Outcomes

Study primary outcome was to evaluate 30-days postoperative complications amongst the two groups.

Secondary endpoints were: LOS and prolonged LOS (defined as any LOS greater 1.5 times the median LOS in matched cohorts), time to tolerated fluid and food intake, time to first defecation, rate of anastomotic leak, abdominal collection, bleeding, strictures, pulmonary complications, surgical site infections, 30-days reoperation, 30-days readmission, 30-days mortality.

### Statistical analysis

Characteristics were summarized by means of the levels for categorical variables or by means of quantiles for continuous variables. Non-parametric tests were performed for comparisons between groups (Chi-Squared and Fisher Exact test in case of categorical variables, Wilcoxon test in case of continuous variables). Cox-Stuart test was used to test whether the data have an increasing or decreasing trend. All tests were 2-sided, accepting p < 0.05 as indicating a statistically significant difference and confidence intervals were calculated at 95% level. The analysis was performed using the R software (R Core Team (2020). R: A language and environment for statistical computing. R Foundation for Statistical Computing, Vienna, Austria. URL: https://www.R-project.org/.

### Ethics

This study was conducted according to the international ethical recommendations on clinical research established by the Helsinki Declaration. The study was conducted in accordance with STROBE criteria (htpp://strobe-statement.org) and registered under clinical trials.gov: NCT05259488 [14].

## Results

### Study population

From January 2014 to December 2021 fifty-one consecutive patients diagnosed with stage I-III gastric cancer were scheduled for LTG.

Seven patients did not meet the inclusion criteria and were excluded from the analysis: Two patients were converted to open surgery and in two cases the procedure was abandoned for intraoperative evidence of peritoneal carcinosis for which other procedures can be advisable14. Three patients were excluded because of pre-operative malnutrition (MNA-SF < 12) and underwent 15 days of IV nutrition before LTG.

Forty-four patients undergoing LTG were subsequently included in the study analysis: 23 patients receiving IN (Group A) were compared to 21 patients operated between January 2014 and December 2017 that had followed standard dietary advice.

**Fig. 2:**
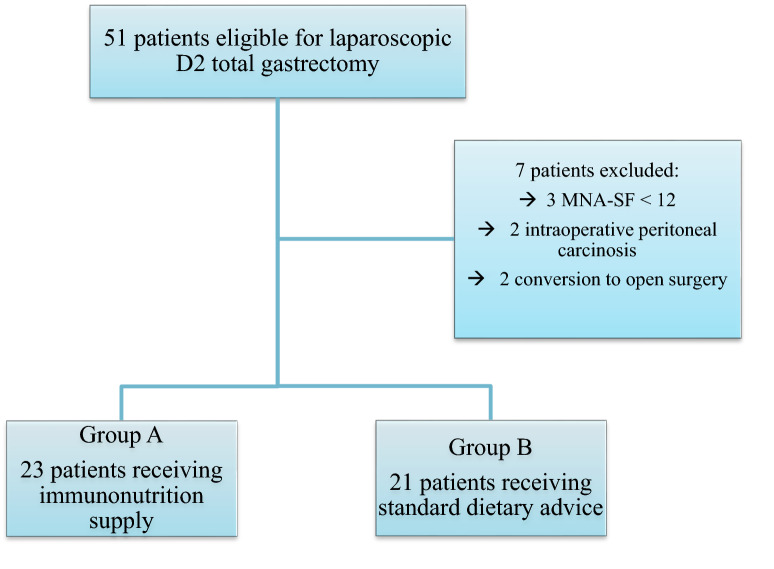
Patient selection

Patient selection is summarized in Fig. 2.

### Baseline characteristics

Baselines patients’ characteristics are summarized in Table 2. The two groups were comparable with respect to sex, BMI, ASA score, comorbidities, preoperative albumin, MNA-SF score, staging, neoadjuvant chemotherapy. Patient’s from group A were older than patients from group B (p < 0.406).

**Table 2:** Baseline characteristics

Parameters	Group A (Immunonutrition) (n = 23)	Group B (Standard) (n = 21)	P
Age (mean, SD)	69.7 ± 9.8	64.3 ± 6.7	0.406
Sex, Male	14, 60.9%	13, 61.9%	1
%, Female	9, 38.1%	8, 37.1%	
Preoperative BMI (mean, SD)	25.6 ± 3.6	25.1 ± 4.1	0.668
ASA score 1	2, 8.7%	1, 4.8%	0.869
% 2	7, 30.4%	7, 33.3%	
3	14, 60.9%	13, 61.9%	
Morbidity %			
Diabetes	6, 26.1%	3, 14.3%	0.461
Hypertension	13, 56.5%	10, 47.7%	0.763
Heart disease	3, 13%	5, 23.8%	0.448
Respiratory disease	1, 4.3%	3, 14.3%	0.334
Preoperative albumin (gr/dl) (mean, SD)	3.87 ± 0.4	3.81 ± 0.4	0.621
MNA-SF score (mean, SD)	13.4 ± 0.5	13.2 ± 0.4	0.153
Staging I	2, 8.7%	2, 9.5%	0.988
% II	7, 30.4%	6, 28.5%	
III	14, 60.9%	13, 61.9%	
Neoadjuvant chemotherapy %	17, 73.9%	13, 61.9%	0.521

### Outcomes

Thirty-day postoperative complications were respectively 8.7% in Group A and 33.3% in Group B (p 0.04).

Mean and median LOS for Group A and B were also significantly different: 7.2 ± 4.4 vs 10.3 ± 5.4 and 7 vs 10 days respectively.

No differences were found between groups in first time to defecation, time to tolerated fluids and food intake, PLOS (any LOS greater than 13 days), anastomotic leaks, abdominal collection, strictures, bleeding, pulmonary complication, SSI, reoperation, readmission and mortality rate.

Postoperative complications occurred in two patients in Group A and seven patients in Group B.

In Group A: one patient had an anastomotic leak and sub-phrenic collection furtherly complicated by pneumonia; treatment consisted of radiological drainage, fasting and IV antibiotics (CD3a). The second patient had a postoperative bleeding and a subsequent collection requiring red cells transfusions and antibiotics (CD2).

In Group B: one patient had an anastomosis leak with a large perigastric collection and bilateral pleural effusion treated with endoscopic stent insertion and radiological drain placement. She developed a second leak at the jejunal-jejunal anastomosis, was taken back to theatre and eventually died of sepsis in ICU (CD5). The second anastomotic leak in group B, was successfully treated with endoscopic stent placement and did not have further complications (CD3a); one bleeding followed by perigastric collection required blood transfusions and IV antibiotics (CD3a), whilst a postoperative intraluminal bleeding was treated with transfusions only (CD2). One pancreatic fistula, complicated by left-sided pneumonia was also treated conservatively with antibiotics (CD2) Two patients in group B were readmitted: one for SSI on specimen extraction site requiring vaacum assisted therapy (CD2) and one because of a subfrenic collection requiring antibiotics and radiological drainage (CD3a).

Results of primary and secondary outcomes are summarized in Table 3.

**Table 3:** Results of primary and secondary outcomes

Parameters	Group A (Immunonutrition) (n = 23)	Group B (Standard) (n = 21)	P
Time to first defecation, days (mean, SD)	3.9 ± 2.5	4.3 ± 2.3	0.599
Time of tolerated fluid intake, days (mean, SD)	2.7 ± 1.9	3.9 ± 3.3	0.113
Time of tolerated food intake, days (mean, SD)	4.8 ± 2.7	6.4 ± 3.8	0.103
Length of stay, days (mean, SD)	7.2 ± 4.4	10.3 ± 5.4	0.04
PLOS	2, 8.7%	5, 23.8%	0.222
30-day complications	2/23, 8.7%	7/21, 33.3%	0.04
Anastomotic leak	1, 4.3%	2, 9.5%	0.589
Anastomosis stricture	0, 0%	0, 0%	1
Abdominal collection	2, 8.7%	3, 14.3%	0.644
Bleeding	1, 4.3%	2, 9.5%	0.589
Pulmonary complication	1, 4.3%	2, 9.5%	0.589
SSI	0, 0%	1, 4.8%	0.465
Reoperation rate	0, 0%	0, 0%	1
30 days readmission rate	0, 0%	2, 9.5%	0.21
30 days mortality rate	0, 0%	1, 4.8%	1

## Discussion

Application of evidence-based perioperative care protocols seems effective in ameliorate outcomes of colorectal, hepatobiliary and upper gastrointestinal surgery [16–19]. Enhanced recovery after surgery (ERAS) has progressed especially in colorectal surgery, but current guidelines point to the lack of evidence in upper gastrointestinal surgery. Since 2017, four meta-analysis have been published evaluating the state of the ERAS protocols in gastric cancer surgery [20–23].

Two papers focused on postoperative outcomes, demonstrating rapid recovery and shorter postoperative stay after laparoscopic gastrectomy [20, 21]. Ding et al. highlighted an improvement of postoperative inflammatory response but increased readmission rate without differences in postoperative complications [20]. An increased readmission rate was also found by Lee et al. and Wee et al., even if LOS, complication rates, return to bowel functions, were lower [24, 25]. Wang et al., in their metanalysis assessed the safety and efficacy of ERAS that led to a reduction of surgical stress and convalescence, and to an improvement of nutritional status [23]. Almost all papers suggested that ERAS is effective in gastric cancer surgery helping reducing costs, shortening LOS and complication rate especially when associated to laparoscopic surgery. However, in particular laparoscopic total gastrectomy (LTG) still represent a great challenge and it is not fully accepted in the common clinical practice even though, in experienced hands, is safe and feasible providing good results [26].

In most protocols of ERAS, large space is dedicated to perioperative nutrition [27–30]. Malnutrition affects negatively the host immune response and is associated with increased morbidity after surgery [31–35]. However, results from studies on perioperative IN are inconsistent and, although a benefit cannot be excluded, a clear evidence is still lacking, especially in norm-nourished patients [6, 27, 33].

IN with arginine, omega 3 fatty acids, nucleotides, glutamine, etc., given in the perioperative period, could reduce the synthesis of pro-inflammatory cytokines, while it could stimulate the production of glutathione, which can decrease oxidative injury. Arginine is a key element in wound and anastomosis healing [36], omega-3 and 6 play an anti-inflammatory role by contrasting oxidative injury, downregulating arachidonic acid. The addition of carbohydrates loading prevents the insulin-resistance due to cortisol and glucagon raised after surgery [37].

Our study focused on preoperative IN in a homogenous group of normo-nourished gastric cancer patients undergoing LTG following an ERAS protocol [38–42]. Weight loss is particularly important for patients with gastric cancer undergoing total gastrectomy and every effort is made to reduce the risk of perioperative malnutrition. Only few reports of IN in patients undergoing LTG have been published [26, 43–45].

To assess patients’ nutritional status, it was used the MNA-SF questionnaire, a 6-questions based malnutrition screening test, capable to individualize malnourished patients but also patients at high-risk of malnutrition.

The rate of severe complication was lower in the group of patients who had preoperative IN compared to the control group (8.7% vs 33.3%) and lower was also the LOS (7.2 vs 10.3 days). Patients in the study group were older than in the control; it is reported that age is a potential conflicting variable to the efficacy of ERAS protocol but in this single centre experience, the level of compliance was high [46, 47]. The fact that all patients in study group were treated accordingly to one single ERAS protocol, implemented with IN and followed up in a single unit, is one of the strengths of this study. A single surgeon has performed all the surgical procedures and this possibly reduce the risk of technical bias. The major limitation of this retrospective study is that is prone to historical bias, caused by change in practice, development of the learning curve and enhancement of peri-operative care. Clearly a randomization was not possible but neither was a selection: patients in both groups, study or control, all underwent LTG with the same technique, following the same oncology protocol. Perioperative care was different amongst the two groups but applied to all patients within its own group. Reduction in complications observed in group A could be attributable to both IN and/or ERAS. Nevertheless, the reduction of LOS could be attributed to both a reduction in complications’ rate, or to a change of perioperative management with the introduction of ERAS.

## Conclusions

The implementation of ERAS and IN seems to reduce LOS and overall complications after LTG. Early feeding is well accepted even in the elderly.

## Data Availability

All data generated or analyzed during this study are included in this published article.
